# Investigating molecular basis of lambda-cyhalothrin resistance in an *Anopheles funestus* population from Senegal

**DOI:** 10.1186/s13071-016-1735-7

**Published:** 2016-08-12

**Authors:** Badara Samb, Lassana Konate, Helen Irving, Jacob M. Riveron, Ibrahima Dia, Ousmane Faye, Charles S. Wondji

**Affiliations:** 1 Laboratoire d’Écologie Vectorielle et Parasitaire, Département de Biologie Animale, Faculté des Sciences et Techniques, Université Cheikh Anta Diop de Dakar, Dakar-Fann, BP 5005 Sénégal; 2 Department of Vector Biology, Liverpool School of Tropical Medicine, Pembroke Place, Liverpool, L3 5QA UK; 3 Research Unit Liverpool School of Tropical Medicine, Organisation de Coordination pour la lutte contre les Endémies en Afrique Centrale, P.O Box 288, Yaoundé, Cameroon; 4 Unité d’Entomologie Médicale, Institut Pasteur de Dakar, 36 Avenue Pasteur, BP 220 Dakar, Sénégal

**Keywords:** *Anopheles funestus*, Insecticide resistance, Lambda-cyhalothrin, Resistance mechanisms, Senegal

## Abstract

**Background:**

*Anopheles funestus* is one of the major malaria vectors in tropical Africa, notably in Senegal. The highly anthropophilic and endophilic behaviours of this mosquito make it a good target for vector control operations through the use of insecticide treated nets, long-lasting insecticide nets and indoor residual spraying. However, little is known about patterns of resistance to insecticides and the underlying resistance mechanisms in field populations of this vector in Senegal.

**Methods:**

Here, we assessed the susceptibility status of *An. funestus* populations from Gankette Balla, located in northern Senegal and investigated the potential resistance mechanisms.

**Results:**

WHO bioassays indicated that *An. funestus* is resistant to lambda-cyhalothrin 0.05 % (74.64 % mortality), DDT 4 % (83.36 % mortality) and deltamethrin 0.05 % (88.53 % mortality). Suspected resistance was observed to permethrin 0.75 % (91.19 % mortality), bendiocarb 0.1 % (94.13 % mortality) and dieldrin 4 % (96.41 % mortality). However, this population is fully susceptible to malathion 5 % (100 % mortality) and fenitrothion 1 % (100 % mortality). The microarray and qRT-PCR analysis indicated that the lambda-cyhalothrin resistance in Gankette Balla is conferred by metabolic resistance mechanisms under the probable control of cytochrome P450 genes among which CYP6M7 is the most overexpressed. The absence of overexpression of the P450 gene, CYP6P9a, indicates that the resistance mechanism in Senegal is different to that observed in southern Africa.

**Conclusions:**

This study represents the first report of pyrethroid and DDT resistance in *An. funestus* from Senegal and shows that resistance to insecticides is not only confined to *An. gambiae* as previously thought. Therefore, urgent action should be taken to manage the resistance in this species to ensure the continued effectiveness of malaria control.

**Electronic supplementary material:**

The online version of this article (doi:10.1186/s13071-016-1735-7) contains supplementary material, which is available to authorized users.

## Background

The burden of malaria remains heaviest in the WHO African region, where an estimated 90 % of all malaria deaths occur, and in children aged under five years, who account for 78 % of all deaths [1]. In Senegal, malaria is a major cause of morbidity and mortality and a high priority for the government, even though the number of reported cases of malaria has dropped significantly since 2007–2008 [2]. As in many African countries, malaria control in Senegal relies heavily on vector control through the use of long-lasting insecticide nets (LLINs) and indoor residual spraying (IRS). However, resistance to the main insecticides in the major malaria vectors such as *Anopheles funestus* is threatening the success of these control interventions. Resistance to different classes of insecticides used in public health is increasingly reported across Africa in *An. funestus* with fear that this could disrupt control programs against this vector. Indeed, resistance to pyrethroids, DDT and carbamates has been reported in different regions of Africa including southern Africa [3–6], Central Africa [7], East Africa [8, 9] and West Africa [10, 11]. In Senegal, pyrethroid resistance until recently was mainly reported in *An. gambiae* [12–14] while little is known about the susceptibility of *An. funestus* to insecticides. Senegal is currently scaling up its malaria control program through LLINs and IRS [15, 16]. It is crucial that information on susceptibility to main insecticides used in public health and the underlying mechanisms be investigated. This will properly inform control programs of the most suitable insecticides to use and facilitate the design of appropriate resistance management strategies. In this study, we report the assessment of the susceptibility of one *An. funestus* population from northern Senegal to several insecticides used in public health and also investigate the underlying molecular mechanisms conferring resistance to lambda-cyhalothrin, a key pyrethroid insecticide used for IRS. This information will fill the gap in our knowledge on the resistance distribution in *An. funestus* and help to improve future control programs on this species in Senegal.

## Methods

### Study site and mosquito collection

Blood-fed *An. funestus* adult females resting indoor were collected in houses between 7.00 am and 15.00 pm in November 2011, in Gankette Balla (15°58′N, 15°55′W), which is located in the Lake Guiers area, in northern Senegal, West Africa. Blood-fed and gravid mosquitoes resting inside houses were collected using aspirators and torches and kept in small cups, covered by a non-treated net, until fully gravid and transported to the insectary where they were allowed to lay eggs [17] and hatch in larvae bowls for rearing. The egg batches were pooled and reared together and the F_1_ adults generated were randomly mixed in cages for subsequent experiments.

### Species identification

All females used for individual oviposition were morphologically identified as belonging to the *An. funestus* group [18]. Genomic DNA was extracted from head and thorax using the Livak protocol as previously described [19]. A cocktail PCR was carried to confirm that all females that laid eggs were *An. funestus* (*s.s.*) [20].

### Adult mosquito susceptibility assays

Insecticide susceptibility assays were carried out using 2–5 day-old F_1_ adults following the WHO protocol [21]. Approximately 20–25 mosquitoes per tube with 4–6 replicates were exposed to insecticide-impregnated filter paper for 1 h or control paper and then transferred to a clean holding tube supplied with 10 % sugar and mortality was determined 24 h post-exposure. We tested the following insecticides: the pyrethroids permethrin (0.75 %), lambda-cyhalothrin (0.05 %) and deltamethrin (0.05 %); the carbamate bendiocarb (0.01 %); the organophosphate malathion (5 %) and fenitrothion (5 %) and the organochlorines DDT (4 %) and dieldrin (4 %).

### Microarray analysis

A custom microarray chip [3] containing 8 × 60 k probes (60mer) (A-MEXP-2374) was used to identify the set of genes associated with lambda-cyhalothrin resistance [22]. RNA was extracted from three batches of 10 *An. funestus* females that were 2–5 day-old from the following sample sets: alive after exposure to 0.75 % lambda-cyhalothrin (R); unexposed to insecticides and thus representative of the wild-type population (C); and unexposed mosquitoes from the fully susceptible laboratory strain FANG (S). RNA was isolated using the Picopure RNA isolation kit (Arcturus, Applied Biosystems, Carlsbad, CA, USA). The quantity and quality of extracted RNA were assessed using a NanoDrop ND1000 spectrophotometer (Thermo Fisher Scientific, Waltham, MA, USA) and a Bioanalyzer (Agilent, Santa Clara, CA, USA), respectively. cRNA of each sample was amplified using the Agilent Quick Amp labeling kit (two-colour) following the manufacturer’s protocol. cRNA from the resistant samples (R) were labelled with cy5 dye and cRNA from the control (C) were labelled with both cy3 and cy5, whereas the susceptible strain FANG (S) was labelled with the cy3 dye. cRNA quantity and quality were checked after labelling using the NanoDrop spectrophotometer and Bioanalyzer. Labelled cRNAs were hybridized to the arrays for 17 h at 65 °C according to the manufacturer’s protocol. Five hybridizations for each of the comparisons (R-S, R-C and C-S) were carried out by swapping the biological replicates. Microarray data were analysed using Genespring GX 12.0 software. To identify differentially expressed genes, a cut-off of 1.5 (R-C) and 2-fold-change (FC) (R-S and C-S) and a statistical significance of P ≤ 0.05 with Benjamin-Hochberg correction for multiple testing were applied.

### Transcription profiling of candidate metabolic resistance genes (qRT-PCR)

The main candidates resistance genes (Additional file 1: Table S1) that were highly overexpressed from the microarray analysis were assessed by quantitative Reverse Transcriptase PCR (qRT-PCR) to validate their expression pattern using three biological replicates (10 females F_1_ for each replicate) for lambda-cyhalothrin resistant mosquitoes (R) (alive after 24 h exposure to lambda-cyhalothrin), control mosquitoes (C) (not exposed to any insecticide) and susceptible FANG mosquitoes (S). One microgram of total RNA from each of the three biological replicates for resistant (R), control (C), and FANG (S) was used as a template for cDNA synthesis using the Super-Script III (Invitrogen, Carlsbad, CA, USA) with oligo-dT20 and RNase H, according to the manufacturer’s instructions. A serial dilution of cDNA was used to establish standard curves for each gene to assess PCR efficiency and quantitative differences between samples. The q-RT-PCR amplification was carried out in a MX3005 real-time PCR system (Agilent, Santa Clara, CA, USA) using Brilliant III Ultra-Fast SYBR Green QPCR Master Mix (Agilent). A total of 10 ng of cDNA from each sample was used as template in a three-step program involving a denaturation at 95 °C for 3 min followed by 40 cycles of 10 s at 95 °C and 10 s at 60 °C and a last step of 1 min at 95 °C, 30 s at 55 °C, and 30 s at 95 °C. The relative expression and fold-change of each target gene in R and C relative to S was calculated according to the 2^−ΔΔCT^ method incorporating PCR efficiency [23] after normalization with the housekeeping *RSP7* (ribosomal protein S7; AFUN007153-RA) and the *Actin* (AFUN006819) genes.

### Investigation of the role of knockdown resistance mutation in lambda-cyhalothrin resistance

A fragment spanning a portion of the voltage-gated sodium channel gene (VGSC), including the 1014 codon associated with resistance in *An. gambiae*, was amplified using the KdrFunF2/KdrFunR2 primers [9, 11, 24] and sequenced (KdrFunR2) in ten field-collected *An. funestus* female mosquitoes from Gankette Balla. PCR, sequencing and analysis were carried out as previously described [9, 11, 24].

## Results

### Mosquito collection

More than 450 blood-fed *An. funestus* females were collected inside houses in the village of Gankette Balla over a period of four days in November 2011. Around 150 laid eggs and all were confirmed to be *An. funestus* (*s.s.*) by PCR.

### Insecticide susceptibility

**Fig. 1:**
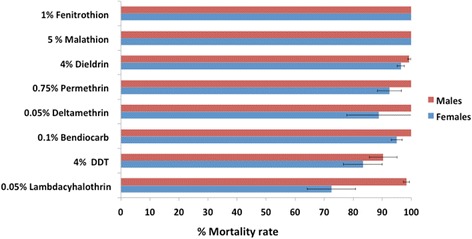
Insecticide resistance profile of *Anopheles funestus* populations from Gankette Balla

A total of 2,322 F_1_*An. funestus* adults from Gankette Balla were generated and exposed to various insecticides (Additional file 2: Table S2). The bioassay performed indicated that the *An. funestus* population females were resistant to the type II pyrethroids lambda-cyhalothrin and deltamethrin as well as to the organochlorine DDT with mortality rates of 74.64 %, 88.53 % and 83.36 %, respectively. Suspected resistance was observed to permethrin, bendiocarb and dieldrin with mortality rates of 91.19 %, 94.13 % and 96.41 %, respectively. However, this population was fully susceptible to the organophosphates malathion and fenitrothion with a mortality rate of 100 %. The males of this *An. funestus* population were generally susceptible to exposed insecticides except for DDT and lambda-cyhalothrin for which a moderate resistance was observed with mortality rates of 90.30 % and 97.30 %, respectively (Fig. 1).

### Genome-wide microarray-based transcription analysis of lambda-cyhalothrin resistance

**Fig. 2:**
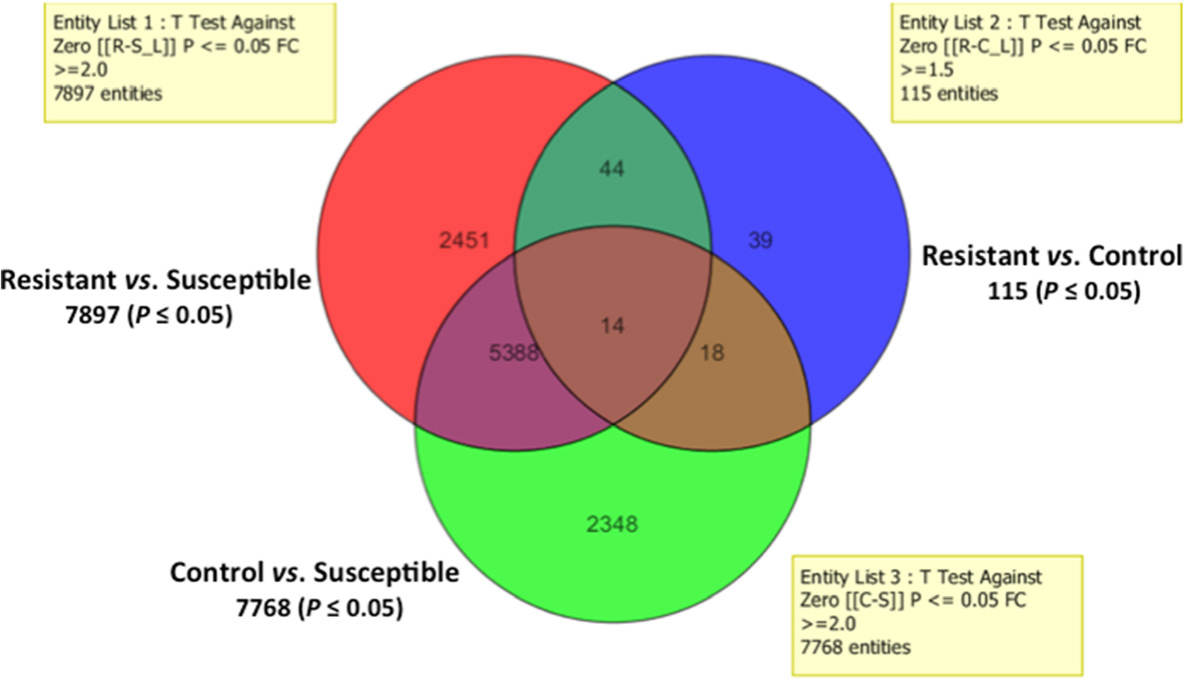
Summary of probes differentially expressed in Lambda-cyhalothrin resistance

The total number of differentially expressed probes for the comparison between lambda-cyhalothrin resistant samples and the susceptible strain FANG (R-S) was 7,897 (3,789 overexpressed); 7,768 (4,055 overexpressed) for the comparison between the control wild type samples (C) and the susceptible strain FANG (C-S); 115 (59 overexpressed) for the comparison between lambda-cyhalothrin resistant samples and the control wild type samples R-C) (Fig. 2). Fourteen probes were commonly differentially expressed in the three types of comparison (R-S, C-S and R-C). However, 5,388 probes were commonly differentially expressed in the R-S and C-S comparisons, whereas 44 and 18 probes were respectively commonly differentially expressed in the pairs of comparisons R-S *vs* R-C and C-S *vs* R-C (Fig. 2).

### Genes commonly overexpressed in R-S, C-S and R-C comparisons

A probe for the CYP6M7 transcript, belonging to the cytochrome P450 gene family, was the most commonly overexpressed detoxification gene in the R-S (FC 101.64) and C-S (FC 120.09) comparisons. This gene was also significantly overexpressed in the R-C comparison but with a much lower FC value (2.59) (Table 1). Other cytochrome P450s genes that were also commonly overexpressed in the three comparisons were three other P450 genes including CYP6AH1 (combined_c1486), CYP304b1 and CYP4C36 (Afun007127). The transcripts Afun008293 and Afun009227 belonging to trypsin-related protease and argininosuccinate lyase genes respectively, were commonly highly overexpressed in R-S and C-S comparisons and significantly overexpressed in R-C. Transcripts belonging to others detoxification genes family such as glutathione transferase (GSTd3), abc transporter (Afun015523), carboxylesterase (Afun011942) were also significantly commonly overexpressed in the three comparisons (Table 1).

**Table 1:** Transcripts from detoxification genes with lambda-cyhalothrin resistance upregulated in various comparisons (R-C, R-S and C-S) in Gankette Balla

Probe name	Transcript	R-C	R-S	C-S	Description
CUST_7663_PI426302897	CYP6M7	2.59	101.64	120.09	cytochrome p450
CUST_2949_PI406199769	combined_c1486 (CYP6AH1)	1.52	5.59	5.27	cytochrome p450
CUST_12197_PI426302897	CYP304b1	1.61	4.44	2.74	cytochrome p450
CUST_7127_PI426302897	Afun007127 (CYP4C36)	2.17	3.23	2.90	cytochrome p450
CUST_8293_PI426302897	Afun008293	1.78	79.77	63.27	trypsin-related protease
CUST_13921_PI426302897	Afun013921	2.21	34.28	25.55	chymotrypsin 1
CUST_8354_PI426302897	GSTD3	1.52	6.33	4.69	glutathione transferase (agap004382-pa)
CUST_7773_PI426302897	Afun007773	1.57	6.06	4.00	microsomal glutathione s-transferase
CUST_9227_PI426302897	Afun009227	1.79	29.78	42.82	argininosuccinate lyase
CUST_15523_PI426302897	Afun015523	1.55	8.39	8.34	abc transporter
CUST_14150_PI426302897	Afun014150	1.64	7.51	4.70	oxidative stress-induced growth
CUST_11942_PI426302897	Afun011942	1.63	3.47	3.20	carboxylesterase
CUST_376_PI406199788	gb-CYP4H25		10.60	4.99	cytochrome p450
CUST_12777_PI426302897	CYP4C27		9.98	21.93	cytochrome p450
CUST_4223_PI426302897	CYP4H17		9.82	4.86	cytochrome p450
CUST_12343_PI426302897	CYP4H17		6.46	3.90	cytochrome p450
CUST_12197_PI426302897	Afun012197 (CYP304B1)		4.44	2.74	cytochrome p450
CUST_7369_PI426302897	Afun007369 (CYP6P9b)		4.04	4.68	cytochrome p450
CUST_9312_PI426302897	Afun009312		25.95	38.54	high affinity gaba transporter
CUST_3489_PI406199769	combined_c1762		4.48	3.52	abc transporter
CUST_5545_PI426302897	Afun005545		14.97	18.72	ankyrin repeat domain protein
CUST_3386_PI426302897	Afun003386		5.50	4.54	ankyrin repeat domain-containing protein 50
CUST_10406_PI426302897	Afun010406		4.15	3.86	ankyrin repeat domain-containing protein 50
CUST_1930_PI426302897	Afun001930		4.39	3.88	ankyrin repeat-containing
CUST_3672_PI426302897	Afun003672		4.35	4.19	multiple ankyrin repeats single kh d. protein
CUST_1459_PI406199769	combined_c738		9.17	10.93	short-chain dehydrogenase
CUST_2520_PI406199772	CD578141.1		5.54	6.72	short-chain dehydrogenase
CUST_10105_PI426302897	Afun010105		3.38	2.44	short-chain dehydrogenase
CUST_12461_PI426302897	Afun012461		8.67	3.72	alcohol dehydrogenase
CUST_665_PI406199788	gb-NADH_dehyd		2.86	4.87	nadh dehydrogenase
CUST_10836_PI426302897	Esterase b1		7.33	3.75	esterase b1
CUST_11942_PI426302897	Afun011942		3.47	3.20	carboxylesterase
CUST_34_PI406199775	COEAE6O		2.41	2.05	carboxylesterase
CUST_13332_PI406199769	combined_c6826		2.06	2.33	esterase b1
CUST_295_PI406199798	AGAP000177-RA		7.06	16.73	cuticle protein 7
CUST_3736_PI406199772	CD577515.1		5.94	3.38	cuticle protein
CUST_8354_PI426302897	GSTD3		6.33	4.69	glutathione transferase (agap004382-pa)
CUST_7773_PI426302897	Afun007773		6.06	4.00	microsomal glutathione s-transferase
CUST_1870_PI406199769	combined_c944		4.25	2.09	microsomal glutathione s-transferase
CUST_10360_PI426302897	Afun010360		2.63	2.84	glucosyl glucuronosyl transferases
CUST_7499_PI426302897	GSTd1-5		2.55	2.48	glutathione transferase
CUST_8698_PI426302897	Afun008698		5.74	25.76	heat shock protein 70 b2
CUST_7498_PI426302897	Afun007498		3.69	2.81	heat shock cognate 70 protein
CUST_7302_PI426302897	Afun007302		3.34	3.86	heat shock 70 kda protein cognate 4
CUST_5336_PI426302897	Afun005336		2.94	4.69	heat shock cognate 70 kda protein
CUST_2701_PI406199769	combined_c1362		2.77	2.35	heat shock protein 70 -interacting protein
CUST_2184_PI406199772	CD578312.1		2.30	3.18	82 kda heat shock protein
CUST_3246_PI426302897	Afun003246		5.12	5.83	aldehyde oxidase
CUST_11963_PI426302897	Afun011963		4.56	4.55	aldehyde oxidase
CUST_718_PI406199788	gb-PX4B		4.32	4.43	oxidase peroxidase
CUST_199_PI426302897	Afun000199		3.04	3.46	chorion peroxidase
CUST_7400_PI426302897	Afun007400		2.57	3.12	thioredoxin-dependent peroxidase
CUST_8347_PI426302897	Afun008347		5.07	3.21	chymotrypsin 1
CUST_11697_PI426302897	Afun011697		3.04	4.34	chymotrypsin bii
CUST_7894_PI426302897	Afun007894		5.50	4.34	trypsin delta gamma
CUST_1313_PI426302897	Afun001313 (CYP9J5)	2.93	2.67		cytochrome p450
CUST_11293_PI426302897	Afun011293	4.22	4.97		AGAP012443-PA [Anopheles gambiae str. PEST]
CUST_4992_PI426302897	Afun004992	3.46	2.61		AGAP010545-PA [Anopheles gambiae str. PEST]
CUST_9542_PI426302897	Afun009542	2.32	2.58		AGAP000321-PA [Anopheles gambiae str. PEST]
CUST_9600_PI406199769	combined_c4862	1.51		2.53	ankyrin unc44

*Abbreviations*: *R-C* Resistant-Control, *R-S* Resistant-Susceptible, *C-S* Control-Susceptible

### Genes commonly overexpressed in R-S and C-S comparisons

Several detoxification genes or resistance-related genes were commonly and significantly overexpressed in the R-S and C-S comparison. A set of five transcripts belonging to cytochrome P450s genes was commonly upregulated in R-S and C-S, with CYP4H25 (FC 10.60; 4.99), CYP4C27 (FC9.98; 21.93) and CYP4H17 (FC9.82; 4.86) being the most overexpressed (Table 1). A transcript (Afun007369) with closest hit to CYP6P9b (92 %) was also overexpressed in both R-S (FC4.04) and C-S (FC4.68). However, none of the three probes designed for CYP6P9a was overexpressed in this population, suggesting a significant difference with southern African populations where this P450 is highly overexpressed. Among the most commonly overexpressed resistance-associated genes in R-S and C-S were the high affinity GABA transporter (Afun009312) and an ankyrin repeat domain protein (Afun005545). Noticeably, the transcripts Afun008698 and AGAP000177-RA belonging to heat shock protein 70 b2 and cuticle protein 7 genes successively were commonly overexpressed with a high FC observed in C-S. Furthermore, several probes from the GSTs gene family were also significantly and commonly overexpressed in R-S and C-S in possible association with the observed DDT resistance in this population. These GST genes include the GSTd1-5 previously also reported in southern African populations of *An. funestus* [4]. However, GSTe2, the major DDT resistance gene observed in West and Central African countries [4] is overexpressed in this Senegalese population. The other detoxification or resistance related genes are reported in Table 1 and Additional file 3: Table S3 and include short-chain dehydrogenase, esterase b1, aldehyde oxidase and chymotrypsin 1.

### Genes commonly overexpressed in R-C and R-S comparisons

Only a limited number of genes were commonly overexpressed in the two comparisons. Indeed, these genes include: a single P450 (CYP9J5), three probes corresponding to *An. gambiae* orthologous genes, one probe for membrane-associated LPS-inducible TNF-alpha factor protein and zinc metalloproteinase nas-12, successively (Table 1). The top 50 of the most detoxification genes overexpressed only in the comparisons R-S_L (Mosquitoes resistant to lambda-cyhalothrin *vs* susceptible mosquitoes) and R-C_L (Mosquitoes resistant to lambda-cyhalothrin *vs* control mosquitoes) are presented in Additional file 4: Tables S4 and Additional file 5: Table S5, respectively.

### Genes commonly overexpressed in R-C and C-S comparisons

Only one transcript (combined_c4862) belonging to ankyrin unc44 gene was commonly overexpressed in R-C (FC 1.51) and C-S (FC 2.53) comparisons (Table 1).

### Genes commonly underexpressed in lambda-cyhalothrin resistant mosquitoes

The detoxifying gene “CD578215.1” (cuticle protein) was the most commonly underexpressed gene in the R-S_L (FC 24.47), C-S (FC 56.22) and R-C_L (FC 1.52) comparisons. Other genes that were commonly underexpressed in these three comparisons include: combined_c3712 (stress-sensitive b), CD577548.1 (cytochrome *c* oxidase subunit 1), CD577574.1 (glutathione s-transferase) (Additional file 6: Table S6). The detoxifying gene “Afun013280” (carboxypeptidase n subunit 2) was the most commonly under expressed gene in the R-S_L (FC 29.52) and C-S (FC 16.58) comparisons. Among the others detoxifying gene families commonly underexpressed in R-S_L and CS, we noted the presence of many probes from cytochrome *c* oxidase subunit III and also from F0 ATP synthase subunit 6 (Additional file 7: Table S7). The top 50 of the most detoxification genes underexpressed only in the comparisons R-S_L and R-C_L are presented in Additional file 8: Table S8 and Additional file 9: Table S9, respectively.

### Validation of the microarray upregulation results by qRT-PCR

**Fig. 3:**
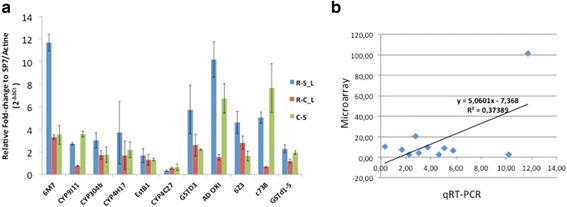
Gene expression analysis. **a** q-RT PCR expression Profile of selected candidate genes in *An. funestus* resistant to lambda-cyhalothrin. **b** Correlation between the microarray and qRT-PCR fold change data for the selected candidates genes

Quantitative reverse transcription PCR (q-RT-PCR) was used to validate the microarray results for 11 of the most overexpressed detoxification genes. These genes include six cytochrome P450s (CYP6M7, CYP4H17, CYP4C27, CYP6Z3, CYP9J11, CYP304b1), one dehydrogenase (Combined_c738), two glutathione transferases (GSTd3, GSTd1-5), one esterase (EstB1) and one aldehyde oxidase (Ald oxi). The qRT-PCR results confirmed significantly the overexpression of candidate genes at different levels. Same to the microarray results, the cytochrome P450 CYP6M7 was the most overexpressed detoxification gene (FC 11.69) in the R-S comparison (Fig. 3a). A significant correlation between the qRT-PCR and microarray results was observed (R^2^ = 0.358, *P* = 0.046) (Fig. 3b).

### Role of knockdown resistance in lambda-cyhalothrin resistance

**Fig. 4:**
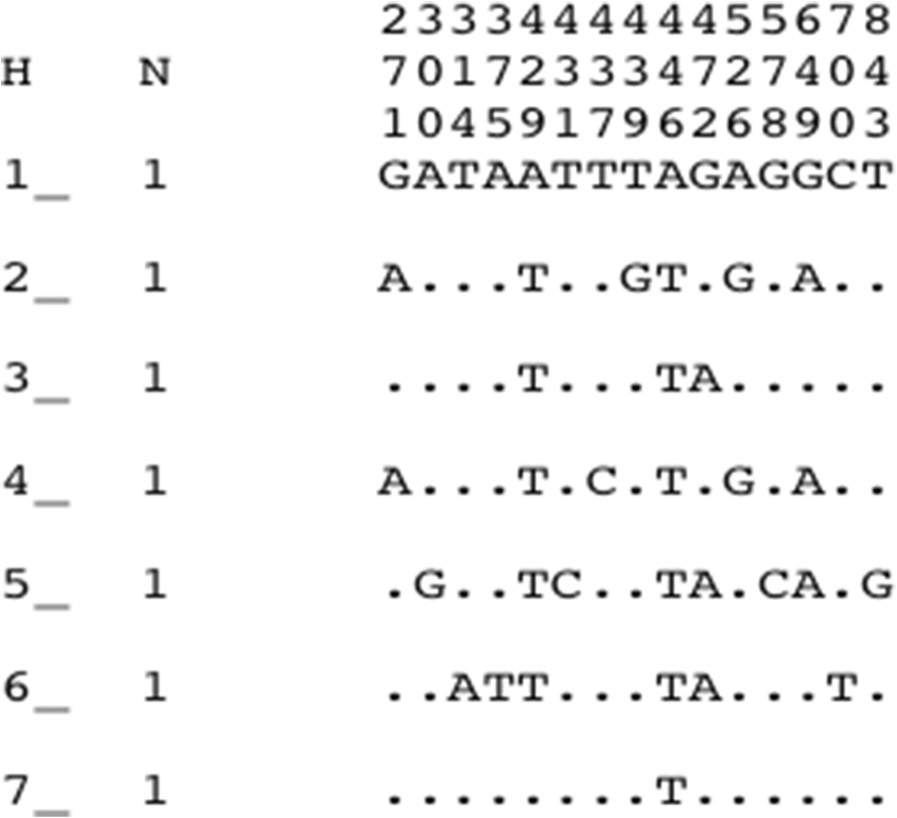
Polymorphic sites at the exon 20 fragment of the VGSC gene in wild type *An. funestus* from Gankette Balla. Only polymorphic sites are shown and these are numbered from the beginning of each aligned sequence. Dots mean identity with the first sequence. A number has been given to each haplotype. The column (N) indicates the number of individuals sharing the haplotype

PCR products were successfully amplified (994 bp) and sequenced for a fragment (a portion of intron 19 and the entire exon 20, domain II, segment 6) of the VGSC gene in ten field-collected *An. funestus* female mosquitoes from Gankette Balla. An 868 bp common sequence was aligned for seven individuals detecting 15 polymorphic sites (Fig. 4). Neither the L1014F *kdr* mutation nor the L1014S mutation was detected in *An. funestus* from Gankette Balla, as previously reported in other populations of this species [9, 11, 25]. Indeed, the VGSC gene sequencing analysis detected only the TTA 1014 codon indicating that they do not have the L1014F (TTA-to-TTT) or L1014S (TTA-to-TCA) kdr mutation (Additional file 10: Figure S1) commonly found in *An. gambiae*.

## Discussion

This study provides the first assessment of the susceptibility and mechanisms to the main insecticides used in public health of *An. funestus* population from Senegal.

### Susceptibility / resistance to lambda-cyhalothrin

The results from this study show that *An. funestus* population from Gankette Balla is resistant to pyrethroids and DDT. In addition, suspected resistance was observed to bendiocarb and dieldrin. Resistance of *An. funestus* to different classes of insecticides has already been reported in West Africa [10, 11, 26], East Africa [9, 17] and in southern Africa [3, 4, 24, 27]. In Senegal, resistance to pyrethroids and DDT has already been reported in other malaria vectors such as *An. gambiae* (*s.l.*) [12–14, 28] and *An. pharoensis* [28].

On the other hand, our study revealed a resistance pattern where type II pyrethroid (lambda-cyhalothrin / deltamethrin) seems to be more involved compared to type I pyrethroids (permethrin). This resistance profile is different to that observed in *An. funestus* in Benin [11] where resistance is higher against type I pyrethroids. This difference could suggest the existence of a different resistance mechanism for pyrethroid resistance in Senegal compared to Benin. Indeed, some cytochrome P450s exhibit specificity for either or the two types of pyrethroids [29, 30]. However, the observed resistance profile is similar to that reported in East Africa [17] and South Africa [31]. The full susceptibility observed for malathion is similar for all tests performed so far on *An. funestus* populations across Africa indicating that this insecticide could be used as an alternative to pyrethroids, carbamates and DDT in Indoor Residual Spraying (IRS) control program directed against this species [32].

The source of the lambda-cyhalothrin resistance observed in Gankette Balla remains unknown although the intense use of this insecticide in the agricultural sector could be a contributing factor. Indeed, due to the permanent availability of water around the Senegal River basin, yearly cultivation is well developed in Gankette Balla and pyrethroids are well represented in the pesticides used. This assumption is reinforced by the fact that the resistance of other malaria vectors to lambda-cyhalothrin was observed in areas of high agricultural pressures in Senegal [28]. Moreover, the link between the resistance of *An. gambiae* (*s.l.*) to insecticides and intensive use of pesticides has been already reported in areas where agriculture is highly developed [33–35].

In addition, another source of selection pressure could be likely linked to the use of type two pyrethroids insecticides (Deltamethrin) in treated bed nets in malaria vector control programs. In fact, the village of Gankette Balla was included during the scaling-up coverage with insecticide-treated nets against malaria in Senegal through the “Pal Fleuve” program in 2006.

Furthermore, the IRS campaign using the lambda-cyhalothrin initiated a year later (2007) in the district of Richard Toll (lower valley of the Senegal River) could have been an additional contributing factor. Indeed, two years after this campaign, a lambda-cyhalothrin resistance in *An. gambiae* (*s.l.*) was observed in this area [28] and a link between this resistance and that observed in our study is not to exclude as previous studies have shown the existence of strong gene flow between populations of *An. funestus* in the lower valley and that of the area of Lake Guiers [36]. The resistance of *An. funestus* to lambda-cyhalothrin and deltamethrin may represent a potential threat to the success of the future vector control program directed against this major vector especially as pyrethroids are the only insecticide used for the impregnation of mosquito nets. This is a serious concern as other vectors of the study area are resistant to pyrethroids [28]. If such resistance is not managed properly, it can still be selected by the current vector control program (Insecticide-treated nets and IRS) to a level that will seriously affect the success of future programs against this major vector. A spread of the observed resistance in Gankette Balla is a concern for the continued effectiveness of pyrethroid-based interventions against *An. funestus* in Senegal.

### Metabolic resistance mechanism is driving lambda-cyhalothrin resistance

Analysis of the transcription profile of the Gankette Balla sample supported the importance of metabolic resistance mechanism in the observed pyrethroid resistance in this *An. funestus* population as previously reported [32]. The importance of metabolic resistance mechanisms is shown through the consistent overexpression of genes involved in insecticide detoxification such as cytochrome P450 genes, GSTs, aldehyde oxidases and other gene families previously associated in resistance of *An. funestus* to insecticides.

The P450 CYP6M7 was consistently the most overexpressed detoxification gene in lambda-cyhalothrin exposed mosquitoes compared to the FANG susceptible strain as well as to the unexposed mosquitoes. This gene was shown to be able to metabolise several pyrethroids including lambda-cyhalothrin [37], further supporting the likelihood that it could be the main resistance gene in this *An. funestus* population. A key role of such P450 gene will be in line with the common implication of the cytochrome P450 family genes in insecticide resistance as previously reported in several populations of *An. funestus* [3, 37–39], *An. gambiae* (*s.l.*) [40], *An. albimanus* [41] and *An. minimus* [42]. This is also reported in several other insects [43–45]. More specifically, the role of cytochrome P450 in pyrethroid resistance has been reported in *An. funestus* from Benin [11], Ghana [10], Uganda [17], Mozambique [24, 27, 46, 47], Kenya [8] and Malawi [3], as well as in the laboratory resistant strain to permethrin [38, 48].

The overexpression of the CYP6M7 gene in lambda-cyhalothrin resistant and non-exposed mosquitoes, compared to the susceptible strain suggests that it plays a key role in pyrethroid metabolic resistance in *An. funestus* in Gankette Balla similar to recent reports for the important role of this gene in Zambia [6, 37]. Surprisingly, one of the duplicated P450 genes, CYP6P9a, which has been shown to play a main role in pyrethroid resistance in southern populations of *An. funestus* [3, 38, 39, 49] was not overexpressed at all. The complete absence of overexpression of CYP6P9a indicates that the resistance mechanism in Senegal is different to that observed in southern Africa. A similar difference is also observed for the GSTe2 gene which is not overexpressed at all in the Gankette Balla population whereas it was among the highest overexpressed in a DDT and pyrethroid resistant population from Benin, another West Africa country. It will be interesting to investigate the role of other GST genes overexpressed in the Gankette Balla population such as GSTd3 and GSTd1-5 to see what role they play in the observed DDT resistance.

Furthermore, the overexpression of several other genes from the microarray analysis suggests that in addition to CYP6M7, other genes may also play a role or involved in subsequent phases of pyrethroid detoxification. Further functional characterisation studies will help to establish these roles.

## Conclusions

This study has provided the first assessment of the susceptibility to the main insecticides used in public health of an *An. funestus* population from Senegal and also explored the possible mechanisms responsible for the pyrethroid resistance observed. The resistance profile observed in the Gankette Balla population highlights the need for further studies to assess the extent and the geographical distribution of these resistances in *An. funestus* populations in Senegal as well as an assessment of its impact on malaria control programs. This will improve the implementation and management of future control programs against this important malaria vector in Senegal and in Africa in general.

## Abbreviations

cDNA, complementary deoxyribonucleic acid; cRNA, complementary ribonucleic acid; DDT, dichlorodiphenyltrichloroethane; DNA, deoxyribonucleic acid; FC, fold change; GABA, gamma-aminobutyric acid; GST, glutathione s-transferase; GSTd1-5, glutathione-s-transferase d1-5; GSTd3, glutathione-s-transferase d3; GSTe2: glutathione-s-transferase epsilon 2; IRS, indoor residual spraying; Kdr, Knockdown resistance; LLINs, long-lasting insecticide nets; PCR, polymerase Chain Reaction; QPCR, real-time quantitative PCR; qRT-PCR, quantitative reverse transcriptase polymerase chain reaction; RNA, ribonucleic acid; RSP7, ribosomal protein S7; VGSC, voltage-gated sodium channel gene; WHO, World Health Organisation

## Additional files

Additional file 1: Table S1. List of primers used in this study. (DOCX 72 kb) Additional file 2: Table S2. Insecticide resistance profile of *Anopheles funestus* population from Senegal. (DOCX 54 kb) Additional file 3: Table S3. Top 50 the most detoxification genes commonly overexpressed in the comparisons R-S_L and C-S (FC ≥2, P ≤ 0.05). (DOCX 108 kb) Additional file 4: Table S4. Top 50 the most detoxification genes overexpressed in the R-S_L comparisons (FC ≥2, P ≤ 0.05). (DOCX 106 kb) Additional file 5: Table S5. Top 50 the most detoxification genes overexpressed in the R-C_L comparisons (FC ≥1.5, P ≤ 0.05). (DOCX 96 kb) Additional file 6: Table S6. Detoxification genes commonly under expressed in the comparisons R-S_L, C-S (FC ≥ 2) and R-C_L (FC ≥ 1.5) P ≤ 0.05. (DOCX 54 kb) Additional file 7: Table S7. Top 50 the most detoxification genes commonly under expressed in the comparisons R-S_L and C-S (FC ≥2, P ≤ 0.05). (DOCX 110 kb) Additional file 8: Table S8. Top 50 the most detoxification genes under expressed in the R-S_L comparisons (FC ≥2, P ≤ 0.05). (DOCX 100 kb) Additional file 9: Table S9. Top 50 the most detoxification genes under expressed in the R-C_L comparisons (FC ≥1.5, P ≤ 0.05). (DOCX 95 kb) Additional file 10: Figure S1. Region flanking the kdr codon 1014 (TTA, indicated in red), showing no variation in wild type *An. funestus* from Gankette Balla. (TIF 1521 kb)
