# Supplementary material for: Investigating molecular basis of lambda-cyhalothrin resistance in an Anopheles funestus population from Senegal
Source: Parasit Vectors. 2016 Aug 12;9:449. doi: 10.1186/s13071-016-1735-7 (PMC4983014; doi:10.1186/s13071-016-1735-7)
Supplement: Additional file 1: Table S1. — List of primers used in this study. (DOCX 72 kb) [file 13071_2016_1735_MOESM1_ESM.docx]

**Table S1:** List of primers used in this study

| **Gene** | **Forward primer** | **Reverse primer** | **Expected size (Pb)** |
| --- | --- | --- | --- |
| **CYP6M7** | CTATCGCCTCAGGGTGGAT | CAGTCGTAACATTATAGCCAAACC | 75 |
| **CYP4H17** | TTGGCCAGAAGTATGCACTG | ATCCGTCTTGTAGCGCACTT | 110 |
| **EstB1** | CCGTTCCATCGGTAGACAAT | GCAGAAGCGATAAACGAAGG | 137 |
| **CYP4C27** | TGGACTGCTCACATCGAAAG | TGCGACTGCAGGTGTTCTAC | 141 |
| **GSTD3F** | CACGGCCAGTCCTCTTTTAG | AAGCTTCTTCGCCACCAGTA | 128 |
| **GSTd1-5F** | TGGAGAAATACGGCAAGGAC | CTTGGCGAAGATTTGTGGAT | 140 |
| **CYP6Z3** | TTTACCCATGCGGATAGAGC | TGGGTTTCCTTTGTACTACACATC | 75 |
| **CYP9J11** | CAAATTTAAAGAGTGCGCTAGG | GTAGATGGTGCCAAGGATGG | 115 |
| **Ald OXI** | GCTCTGAACATTGCACCTCA | TGGTGTCGAACGATTGTGTT | 109 |
| **CYP304b1** | CCGTTTTTGGGTGATTATGG | CACGATCGATGGGAAGTTTT | 132 |
| **Combined_c738** | GCAGGTGACCCATAGTCGTT | GCTGTTGAGGGAATGTTACGA | 136 |
| **RSP7** | GTGTTCGGTTCCAAGGTGAT | TCCGAGTTCATTTCCAGCTC | 98 |
| **Actin** | TTAAACCCAAAAGCCAATCG | ACCGGATGCATACAGTGACA | 111 |
| **KdrFunF2/R2** | GTT CAA TGA AGC CCC TCA AA | CCG AAA TTT GAC AAA AGC AAA | 994 |
