# Supplementary material for: Investigating molecular basis of lambda-cyhalothrin resistance in an Anopheles funestus population from Senegal
Source: Parasit Vectors. 2016 Aug 12;9:449. doi: 10.1186/s13071-016-1735-7 (PMC4983014; doi:10.1186/s13071-016-1735-7)
Supplement: Additional file 2: Table S2. — Insecticide resistance profile of Anopheles funestus population from Senegal. (DOCX 54 kb) [file 13071_2016_1735_MOESM2_ESM.docx]

| **Insecticides** | **Females** | | **Males** | | **Total** | |
| --- | --- | --- | --- | --- | --- | --- |
|  | n | % mortality | n | % mortality | n | % mortality |
| Lambda-cyhalothrin (0.05%) | 222 | 74.64 ±6.54 | 226 | 97.30 ±0.99 | 448 | 85.93 ±4.23 |
| Permethrin (0.75%) | 139 | 91.19 ±2.82 | 129 | 100 ±0 | 268 | 95.52 ±1.98 |
| Deltamethrin (0.05%) | 114 | 88.53 ±7.18 | 117 | 100 ±0 | 231 | 93.93 ±3.89 |
| DDT (4%) | 158 | 83.36 ±6.66 | 98 | 90.32 ±4.78 | 256 | 88.28 ±4.32 |
| Dieldrin (4%) | 306 | 96.41 ±1.26 | 290 | 99.28 ±0.49 | 596 | 97.81 ±0.70 |
| Bendiocarb (0.1%) | 157 | 94.13 ±1.91 | 154 | 100 ±0 | 311 | 97.10 ±1.22 |
| Fenitrothion (1%) | 55 | 100 ±0 | 54 | 100 ±0 | 109 | 100 ±0 |
| Malathion (5%) | 50 | 100 ±0 | 53 | 100 ±0 | 103 | 100 ±0 |

**Table S2:** Insecticide resistance profile of *Anopheles funestus* population from Senegal
