# Supplementary material for: Investigating molecular basis of lambda-cyhalothrin resistance in an Anopheles funestus population from Senegal
Source: Parasit Vectors. 2016 Aug 12;9:449. doi: 10.1186/s13071-016-1735-7 (PMC4983014; doi:10.1186/s13071-016-1735-7)
Supplement: Additional file 3: Table S3. — Top 50 the most detoxification genes commonly overexpressed in the comparisons R-S_L and C-S (FC ≥2, P ≤ 0.05). (DOCX 108 kb) [file 13071_2016_1735_MOESM3_ESM.docx]

**Table S3:** Top 50 the most detoxification genes commonly over expressed in the comparisons **R-S_L** and **C-S** (FC ≥2, P≤ 0.05)

| **Probes Names** | **Transcripts** | **FC Abs R-S_L** | **FC Abs C-S** | **Description** |
| --- | --- | --- | --- | --- |
| CUST_7663_PI426302897 | CYP6M7 | 101.64 | 120.09 | cytochrome p450 |
| CUST_8293_PI426302897 | Afun008293 | 79.77 | 63.27 | trypsin-related protease |
| CUST_13921_PI426302897 | Afun013921 | 34.28 | 25.55 | chymotrypsin 1 |
| CUST_9227_PI426302897 | Afun009227 | 29.78 | 42.82 | argininosuccinate lyase |
| CUST_9312_PI426302897 | Afun009312 | 25.95 | 38.54 | af141930_1high affinity gaba transporter |
| CUST_5545_PI426302897 | Afun005545 | 14.97 | 18.72 | ankyrin repeat domain protein |
| CUST_376_PI406199788 | gb-CYP4H25 | 10.60 | 4.99 | cytochrome p450 |
| CUST_12777_PI426302897 | CYP4C27 | 9.98 | 21.93 | cytochrome p450 |
| CUST_4223_PI426302897 | CYP4H17 | 9.82 | 4.86 | cytochrome p450 4d1 |
| CUST_1459_PI406199769 | combined_c738 | 9.17 | 10.93 | short-chain dehydrogenase |
| CUST_12461_PI426302897 | Afun012461 | 8.67 | 3.72 | alcohol dehydrogenase |
| CUST_310_PI406199798 | AGAP000260-RA___X | 7.70 | 10.42 | atp synthase subunit mitochondrial |
| CUST_14150_PI426302897 | Afun014150 | 7.51 | 4.70 | oxidative stress-induced growth |
| CUST_10836_PI426302897 | Esterase b1 | 7.33 | 3.75 | esterase b1 |
| CUST_295_PI406199798 | AGAP000177-RA | 7.06 | 16.73 | cuticle protein 7 |
| CUST_12343_PI426302897 | CYP4H17 | 6.46 | 3.90 | cytochrome p450 4d1 |
| CUST_8354_PI426302897 | Gstd3 | 6.33 | 4.69 | glutathione transferase (agap004382-pa) |
| CUST_7773_PI426302897 | Afun007773 | 6.06 | 4.00 | microsomal glutathione s-transferase |
| CUST_3736_PI406199772 | CD577515.1 | 5.94 | 3.38 | cuticle protein |
| CUST_8698_PI426302897 | Afun008698 | 5.74 | 25.76 | heat shock protein 70 b2 |
| CUST_2949_PI406199769 | combined_c1486 | 5.59 | 5.27 | cytochrome p450 |
| CUST_2520_PI406199772 | CD578141.1 | 5.54 | 6.72 | short-chain dehydrogenase |
| CUST_3386_PI426302897 | Afun003386 | 5.50 | 4.54 | ankyrin repeat domain-containing protein 50 |
| CUST_3246_PI426302897 | Afun003246 | 5.12 | 5.83 | aldehyde oxidase |
| CUST_8347_PI426302897 | Afun008347 | 5.07 | 3.21 | chymotrypsin 1 |
| CUST_9601_PI406199769 | combined_c4862 | 4.76 | 3.53 | ankyrin unc44 |
| CUST_3489_PI406199769 | combined_c1762 | 4.48 | 3.52 | abc transporter |
| CUST_12197_PI426302897 | Afun012197 | 4.44 | 2.74 | cytochrome p450 |
| CUST_1930_PI426302897 | Afun001930 | 4.39 | 3.88 | ankyrin repeat-containing |
| CUST_3672_PI426302897 | Afun003672 | 4.35 | 4.19 | multiple ankyrin repeats single kh d. protein |
| CUST_718_PI406199788 | gb-PX4B | 4.32 | 4.43 | oxidase peroxidase |
| CUST_1870_PI406199769 | combined_c944 | 4.25 | 2.09 | microsomal glutathione s-transferase |
| CUST_7369_PI426302897 | Afun007369 | 4.04 | 4.68 | cytochrome p450 |
| CUST_7498_PI426302897 | Afun007498 | 3.69 | 2.81 | heat shock cognate 70 protein |
| CUST_11942_PI426302897 | Afun011942 | 3.47 | 3.20 | carboxylesterase |
| CUST_10105_PI426302897 | Afun010105 | 3.38 | 2.44 | short-chain dehydrogenase |
| CUST_7302_PI426302897 | Afun007302 | 3.34 | 3.86 | heat shock 70 kda protein cognate 4 |
| CUST_7127_PI426302897 | Afun007127 | 3.23 | 2.90 | cytochrome p450 |
| CUST_11697_PI426302897 | Afun011697 | 3.04 | 4.34 | chymotrypsin bii |
| CUST_199_PI426302897 | Afun000199 | 3.04 | 3.46 | chorion peroxidase |
| CUST_5336_PI426302897 | Afun005336 | 2.94 | 4.69 | heat shock cognate 70 kda protein |
| CUST_665_PI406199788 | gb-NADH_dehyd | 2.86 | 4.87 | nadh dehydrogenase |
| CUST_2701_PI406199769 | combined_c1362 | 2.77 | 2.35 | heat shock protein 70 -interacting protein |
| CUST_10360_PI426302897 | Afun010360 | 2.63 | 2.84 | glucosyl glucuronosyl transferases |
| CUST_7400_PI426302897 | Afun007400 | 2.57 | 3.12 | thioredoxin-dependent peroxidase |
| CUST_7499_PI426302897 | GSTd1-5 | 2.55 | 2.48 | glutathione transferase |
| CUST_34_PI406199775 | COEAE6O | 2.41 | 2.05 | carboxylesterase |
| CUST_2184_PI406199772 | CD578312.1 | 2.30 | 3.18 | 82 kda heat shock protein |
| CUST_738_PI406199769 | combined_c371 | 2.29 | 4.34 | cytochrome c oxidase subunit iii |
| CUST_13332_PI406199769 | combined_c6826 | 2.06 | 2.33 | esterase b1 |
