# Supplementary material for: Investigating molecular basis of lambda-cyhalothrin resistance in an Anopheles funestus population from Senegal
Source: Parasit Vectors. 2016 Aug 12;9:449. doi: 10.1186/s13071-016-1735-7 (PMC4983014; doi:10.1186/s13071-016-1735-7)
Supplement: Additional file 4: Table S4. — Top 50 the most detoxification genes overexpressed in the R-S_L comparisons (FC ≥2, P ≤ 0.05). (DOCX 106 kb) [file 13071_2016_1735_MOESM4_ESM.docx]

**Table S4:** Top 50 the most detoxification genes overexpressed in the **R-S_L** comparisons (FC ≥2, P≤ 0.05)

| **Probes Names** | **Transcripts** | **FC Abs R-S_L** | **Description** |
| --- | --- | --- | --- |
| CUST_7663_PI426302897 | Afun007663 | 101.64 | cytochrome p450 6a8 |
| CUST_8293_PI426302897 | Afun008293 | 79.77 | trypsin-related protease |
| CUST_13921_PI426302897 | Afun013921 | 34.28 | chymotrypsin 1 |
| CUST_9227_PI426302897 | Afun009227 | 29.78 | argininosuccinate lyase |
| CUST_9312_PI426302897 | Afun009312 | 25.95 | af141930_1high affinity gaba transporter |
| CUST_13273_PI406199769 | combined_c6791 | 20.72 | cytochrome p450 |
| CUST_5545_PI426302897 | Afun005545 | 14.97 | ankyrin repeat domain protein |
| CUST_376_PI406199788 | gb-CYP4H25 | 10.60 | cytochrome p450 |
| CUST_12777_PI426302897 | Afun012777 | 9.98 | cytochrome p450 |
| CUST_4223_PI426302897 | Afun004223 | 9.82 | cytochrome p450 4d1 |
| CUST_1459_PI406199769 | combined_c738 | 9.17 | short-chain dehydrogenase |
| CUST_12461_PI426302897 | Afun012461 | 8.67 | alcohol dehydrogenase |
| CUST_15523_PI426302897 | Afun015523 | 8.39 | abc transporter |
| CUST_310_PI406199798 | AGAP000260-RA | 7.70 | atp synthase subunit mitochondrial |
| CUST_14150_PI426302897 | Afun014150 | 7.51 | oxidative stress-induced growth |
| CUST_10836_PI426302897 | Afun010836 | 7.33 | esterase b1 |
| CUST_295_PI406199798 | AGAP000177-RA | 7.06 | cuticle protein 7 |
| CUST_12343_PI426302897 | Afun012343 | 6.46 | cytochrome p450 4d1 |
| CUST_7674_PI426302897 | Afun007674 | 6.34 | late trypsin |
| CUST_8354_PI426302897 | Afun008354 | 6.33 | glutathione transferase (agap004382-pa) |
| CUST_7773_PI426302897 | Afun007773 | 6.06 | microsomal glutathione s-transferase |
| CUST_3736_PI406199772 | CD577515.1 | 5.94 | cuticle protein |
| CUST_8698_PI426302897 | Afun008698 | 5.74 | heat shock protein 70 b2 |
| CUST_2949_PI406199769 | combined_c1486 | 5.59 | cytochrome p450 |
| CUST_7894_PI426302897 | Afun007894 | 5.50 | trypsin delta gamma |
| CUST_2431_PI426302897 | Afun002431 | 5.18 | stress-activated protein kinase jnk |
| CUST_3246_PI426302897 | Afun003246 | 5.12 | aldehyde oxidase |
| CUST_3723_PI426302897 | Afun003723 | 5.05 | atp-binding cassette sub-family a member 3 |
| CUST_7262_PI406199798 | AGAP005967-RA | 4.94 | adult cuticle |
| CUST_14467_PI426302897 | Afun014467 | 4.87 | glutaminyl-peptide cyclotransferase |
| CUST_55_PI426302897 | Afun000055 | 4.78 | try1_anoga ame: full=trypsin- |
| CUST_9601_PI406199769 | combined_c4862 | 4.76 | ankyrin unc44 |
| CUST_242_PI406199788 | gb-CYP306A1 | 4.64 | cytochrome p450 306a1 |
| CUST_5037_PI426302897 | Afun005037 | 4.62 | n-acetyl galactosaminyl transferase |
| CUST_4924_PI406199798 | AGAP003309-RB | 4.58 | odorant binding protein |
| CUST_12197_PI426302897 | Afun012197 | 4.44 | cytochrome p450 |
| CUST_1930_PI426302897 | Afun001930 | 4.39 | ankyrin repeat-containing |
| CUST_3672_PI426302897 | Afun003672 | 4.35 | multiple ankyrin repeats single kh domain protein |
| CUST_12528_PI426302897 | Afun012528 | 4.33 | voltage-dependent p q type calcium channel |
| CUST_718_PI406199788 | gb-PX4B | 4.32 | oxidase peroxidase |
| CUST_2948_PI406199769 | combined_c1486 | 4.24 | cytochrome p450 |
| CUST_10406_PI426302897 | Afun010406 | 4.15 | ankyrin repeat domain-containing protein 50 |
| CUST_10614_PI426302897 | Afun010614 | 4.11 | atp-binding cassette sub-family a member |
| CUST_11349_PI426302897 | Afun011349 | 4.09 | nadph fad oxidoreductase |
| CUST_7369_PI426302897 | Afun007369 | 4.04 | cytochrome p450 |
| CUST_6021_PI406199769 | combined_c3045 | 3.94 | glucose dehydrogenase |
| CUST_7498_PI426302897 | Afun007498 | 3.69 | heat shock cognate 70 protein |
| CUST_662_PI406199788 | gb-NADH_b5 | 3.52 | nadh-cytochrome b5 reductase |
| CUST_798_PI426302897 | Afun000798 | 3.48 | cytochrome p450 6a8 |
| CUST_11942_PI426302897 | Afun011942 | 3.47 | carboxylesterase |
