# Supplementary material for: Investigating molecular basis of lambda-cyhalothrin resistance in an Anopheles funestus population from Senegal
Source: Parasit Vectors. 2016 Aug 12;9:449. doi: 10.1186/s13071-016-1735-7 (PMC4983014; doi:10.1186/s13071-016-1735-7)
Supplement: Additional file 5: Table S5. — Top 50 the most detoxification genes overexpressed in the R-C_L comparisons (FC ≥1.5, P ≤ 0.05). (DOCX 96 kb) [file 13071_2016_1735_MOESM5_ESM.docx]

**Table S5:** Top 50 the most detoxification genes overexpressed in the **R-C_L** comparisons (FC ≥1.5, P≤0.05)

| **Probes Names** | **Transcripts** | **FC Abs**  **R-C_L** | **Description** |
| --- | --- | --- | --- |
|  |  |  |  |
| CUST_10391_PI426302897 | Afun010391 | 12.59 | dimethylaniline monooxygenase |
| CUST_1410_PI406199769 | combined_c714 | 11.74 | Unknown |
| CUST_3607_PI406199772 | CD577579.1 | 9.59 | Unknown |
| CUST_3608_PI406199772 | CD577579.1 | 9.32 | Unknown |
| CUST_1411_PI406199769 | combined_c714 | 7.49 | Unknown |
| CUST_1269_PI426302897 | Afun001269 | 5.38 | Unknown |
| CUST_3706_PI406199772 | CD577530.1 | 4.88 | Unknown |
| CUST_5387_PI406199769 | combined_c2727 | 4.75 | Unknown |
| CUST_4524_PI406199769 | combined_c2288 | 4.51 | tubulin beta |
| CUST_4525_PI406199769 | combined_c2288 | 4.46 | tubulin beta |
| CUST_5386_PI406199769 | combined_c2727 | 4.37 | Unknown |
| CUST_11293_PI426302897 | Afun011293 | 4.23 | anopheles gambiae pest agap012443-pa |
| CUST_86_PI406199769 | combined_c44 | 4.07 | maltase-like protein agm2 |
| CUST_15277_PI406199769 | combined_c8118 | 3.69 | Unknown |
| CUST_7866_PI406199769 | combined_c3983 | 3.52 | Unknown |
| CUST_7867_PI406199769 | combined_c3983 | 3.52 | Unknown |
| CUST_4992_PI426302897 | Afun004992 | 3.47 | AGAP010545-PA [Anopheles gambiae str. PEST] |
| CUST_9352_PI426302897 | Afun009352 | 3.44 | membrane-associated lps-inducible tnf alpha fact. protein |
| CUST_11306_PI426302897 | Afun011306 | 3.43 | sodium shloride dependent amino acid transporter |
| CUST_7584_PI426302897 | Afun007584 | 3.09 | alpha-amylase |
| CUST_1313_PI426302897 | Afun001313 | 2.94 | cytochrome p450 |
| CUST_1699_PI406199772 | EE589425.1 | 2.92 | d7-related 1 protein |
| CUST_5566_PI426302897 | Afun005566 | 2.88 | Unknown |
| CUST_15754_PI406199769 | combined_c8359 | 2.85 | maltase-like protein agm2 |
| CUST_2124_PI406199769 | combined_c1073 | 2.80 | isoform a |
| CUST_7692_PI426302897 | Afun007692 | 2.73 | gelsolin precursor |
| CUST_9530_PI426302897 | Afun009530 | 2.72 | dual specificity protein phosphatase |
| CUST_2232_PI426302897 | Afun002232 | 2.72 | Unknown |
| CUST_6920_PI426302897 | Afun006920 | 2.47 | stripe-b-like protein |
| CUST_9106_PI406199769 | combined_c4613 | 2.38 | Unknown |
| CUST_9542_PI426302897 | Afun009542 | 2.33 | AGAP000321-PA [Anopheles gambiae str. PEST] |
| CUST_9131_PI426302897 | Afun009131 | 2.30 | delta - |
| CUST_7628_PI426302897 | Afun007628 | 2.13 | isoform b |
| CUST_10929_PI426302897 | Afun010929 | 2.07 | hypothetical conserved protein |
| CUST_8259_PI406199769 | combined_c4182 | 2.07 | Unknown |
| CUST_830_PI426302897 | Afun000830 | 2.05 | gag capsid-like protein |
| CUST_8258_PI406199769 | combined_c4182 | 2.01 | Unknown |
| CUST_14059_PI426302897 | Afun014059 | 1.95 | zinc metalloproteinase nas-12 |
| CUST_11255_PI426302897 | Afun011255 | 1.92 | kda salivary secreted peptide |
| CUST_9743_PI426302897 | Afun009743 | 1.89 | herpud family member 2 |
| CUST_11868_PI426302897 | Afun011868 | 1.89 | acid-sensitive two pore domain k+ channel dtask-7 |
| CUST_2836_PI426302897 | Afun002836 | 1.88 | Unknown |
| CUST_13952_PI426302897 | Afun013952 | 1.84 | atx1_rat ame: full=ataxin-1 ame: full |
| CUST_2820_PI426302897 | Afun002820 | 1.81 | Unknown |
| CUST_14468_PI406199769 | combined_c7573 | 1.77 | Unknown |
| CUST_9862_PI426302897 | Afun009862 | 1.76 | amp dependent ligase |
| CUST_5483_PI406199769 | combined_c2775 | 1.76 | Unknown |
| CUST_5482_PI406199769 | combined_c2775 | 1.75 | Unknown |
| CUST_12574_PI406199769 | combined_c6412 | 1.72 | Unknown |
| CUST_11856_PI426302897 | Afun011856 | 1.72 | ganglioside induced differentiation associated protein |
