# Supplementary material for: Investigating molecular basis of lambda-cyhalothrin resistance in an Anopheles funestus population from Senegal
Source: Parasit Vectors. 2016 Aug 12;9:449. doi: 10.1186/s13071-016-1735-7 (PMC4983014; doi:10.1186/s13071-016-1735-7)
Supplement: Additional file 6: Table S6. — Detoxification genes commonly under expressed in the comparisons R-S_L, C-S (FC ≥ 2) and R-C_L (FC ≥ 1.5) P ≤ 0.05. (DOCX 54 kb) [file 13071_2016_1735_MOESM6_ESM.docx]

**Table S6 :** Detoxification genes commonly under expressed in the comparisons **R-S_L**, **C-S** (FC ≥ 2) and **R-C_L** (FC ≥ 1.5) P ≤ 0.05

| **Probes Names** | **Transcripts** | **FC Abs R-S_L** | **FC Abs C-S** | **FC Abs R-C_L** | **Description** |  |
| --- | --- | --- | --- | --- | --- | --- |
| CUST_2375_PI406199772 | CD578215.1 | 24.47 | 56.22 | 1.51 | cuticle protein | |
| CUST_770_PI406199769 | combined_c387 | 9.48 | 6.43 | 1.50 | mitochondrial cytochrome c oxidase subunit 5b isoform 1 | |
| CUST_7333_PI406199769 | combined_c3712 | 8.29 | 20.39 | 1.54 | stress-sensitive b | |
| CUST_3669_PI406199772 | CD577548.1 | 4.60 | 5.43 | 1.96 | cytochrome oxidase subunit 1 | |
| CUST_3618_PI406199772 | CD577574.1 | 3.87 | 5.20 | 1.83 | glutathione s-transferase | |
| CUST_3173_PI406199772 | CD577811.1 | 3.77 | 5.99 | 1.57 | nadh dehydrogenase | |
