# Supplementary material for: Investigating molecular basis of lambda-cyhalothrin resistance in an Anopheles funestus population from Senegal
Source: Parasit Vectors. 2016 Aug 12;9:449. doi: 10.1186/s13071-016-1735-7 (PMC4983014; doi:10.1186/s13071-016-1735-7)
Supplement: Additional file 7: Table S7. — Top 50 the most detoxification genes commonly under expressed in the comparisons R-S_L and C-S (FC ≥2, P ≤ 0.05). (DOCX 110 kb) [file 13071_2016_1735_MOESM7_ESM.docx]

**Table S7:** Top 50 the most detoxification genes commonly under expressed in the comparisons **R-S_L** and **C-S** (FC ≥2, P≤ 0.05)

| **Probes Names** | **Transcripts** | **FC Abs R-S_L** | **FC Abs C-S** | **Description** |
| --- | --- | --- | --- | --- |
| CUST_13280_PI426302897 | Afun013280 | 29.52 | 16.58 | carboxypeptidase n subunit 2 |
| CUST_2375_PI406199772 | CD578215.1 | 24.47 | 56.22 | cuticle protein |
| CUST_5686_PI406199769 | combined_c2878 | 17.80 | 31.49 | nadh dehydrogenase |
| CUST_4339_PI406199772 | CD577197.1 | 17.72 | 8.33 | cytochrome c oxidase subunit iii |
| CUST_4348_PI406199772 | CD577192.1 | 17.47 | 12.27 | cytochrome c oxidase subunit iii |
| CUST_10482_PI426302897 | Afun010482 | 16.92 | 70.96 | cuticle protein |
| CUST_2538_PI406199772 | CD578132.1 | 16.23 | 22.15 | pupal cuticle protein |
| CUST_4360_PI406199772 | CD577186.1 | 15.93 | 22.05 | cytochrome c oxidase subunit iii |
| CUST_4336_PI406199772 | CD577199.1 | 13.76 | 10.25 | cytochrome c oxidase subunit iii |
| CUST_4385_PI406199772 | CD577173.1 | 13.74 | 13.56 | cytochrome c oxidase subunit iii |
| CUST_4346_PI406199772 | CD577193.1 | 13.38 | 9.73 | cytochrome c oxidase subunit iii |
| CUST_4355_PI406199772 | CD577188.1 | 13.04 | 16.45 | cytochrome c oxidase subunit iii |
| CUST_4508_PI406199772 | CD577111.1 | 13.02 | 11.28 | atp synthase f0 subunit 6 |
| CUST_4559_PI406199772 | CD577084.1 | 12.99 | 9.14 | atp synthase f0 subunit 6 |
| CUST_4376_PI406199772 | CD577178.1 | 12.83 | 12.62 | cytochrome c oxidase subunit iii |
| CUST_4374_PI406199772 | CD577179.1 | 12.68 | 13.01 | cytochrome c oxidase subunit iii |
| CUST_4362_PI406199772 | CD577185.1 | 12.60 | 12.33 | cytochrome c oxidase subunit iii |
| CUST_4334_PI406199772 | CD577200.1 | 12.58 | 7.57 | cytochrome c oxidase subunit iii |
| CUST_4372_PI406199772 | CD577180.1 | 12.51 | 10.59 | cytochrome c oxidase subunit iii |
| CUST_4379_PI406199772 | CD577176.1 | 12.40 | 11.35 | cytochrome c oxidase subunit iii |
| CUST_4337_PI406199772 | CD577198.1 | 12.39 | 7.61 | cytochrome c oxidase subunit iii |
| CUST_1300_PI406199769 | combined_c659 | 12.22 | 8.14 | cytochrome c oxidase subunit iii |
| CUST_1548_PI406199772 | EE589590.1 | 12.21 | 11.06 | cytochrome c oxidase subunit iii |
| CUST_4368_PI406199772 | CD577182.1 | 12.20 | 14.25 | cytochrome c oxidase subunit iii |
| CUST_4396_PI406199772 | CD577168.1 | 12.09 | 12.05 | cytochrome c oxidase subunit iii |
| CUST_4369_PI406199772 | CD577181.1 | 12.07 | 10.27 | cytochrome c oxidase subunit iii |
| CUST_4364_PI406199772 | CD577184.1 | 11.98 | 13.78 | cytochrome c oxidase subunit iii |
| CUST_4366_PI406199772 | CD577183.1 | 11.87 | 12.29 | cytochrome c oxidase subunit iii |
| CUST_4394_PI406199772 | CD577169.1 | 11.86 | 11.03 | cytochrome c oxidase subunit iii |
| CUST_7332_PI406199769 | combined_c3712 | 11.85 | 22.41 | stress-sensitive b |
| CUST_4606_PI406199772 | CD577061.1 | 11.82 | 9.38 | atp synthase f0 subunit 6 |
| CUST_4585_PI406199772 | CD577071.1 | 11.73 | 11.15 | atp synthase f0 subunit 6 |
| CUST_4344_PI406199772 | CD577194.1 | 11.72 | 10.09 | cytochrome c oxidase subunit iii |
| CUST_4384_PI406199772 | CD577174.1 | 11.62 | 10.76 | cytochrome c oxidase subunit iii |
| CUST_4382_PI406199772 | CD577175.1 | 11.44 | 12.28 | cytochrome c oxidase subunit iii |
| CUST_15633_PI406199769 | combined_c8299 | 11.43 | 6.72 | nadh dehydrogenase subunit 5 |
| CUST_4388_PI406199772 | CD577172.1 | 11.43 | 11.64 | cytochrome c oxidase subunit iii |
| CUST_15746_PI406199769 | combined_c8355 | 11.40 | 12.18 | nadh dehydrogenase subunit 5 |
| CUST_2689_PI406199772 | CD578056.1 | 11.36 | 10.72 | nadh dehydrogenase iron-s. p. mitochondrial |
| CUST_4584_PI406199772 | CD577072.1 | 11.12 | 8.78 | atp synthase f0 subunit 6 |
| CUST_21_PI426302915 | CYP6S2.seq | 11.11 | 10.96 | cytochrome p450 |
| CUST_4377_PI406199772 | CD577177.1 | 11.01 | 10.53 | cytochrome c oxidase subunit iii |
| CUST_4392_PI406199772 | CD577170.1 | 11.01 | 10.20 | cytochrome c oxidase subunit iii |
| CUST_4528_PI406199772 | CD577101.1 | 11.00 | 7.71 | atp synthase f0 subunit 6 |
| CUST_10684_PI426302897 | Afun010684 | 10.84 | 2.06 | juvenile hormone esterase |
| CUST_1334_PI406199772 | EE589817.1 | 10.84 | 9.21 | atp synthase f0 subunit 6 |
| CUST_4524_PI406199772 | CD577103.1 | 10.61 | 9.73 | atp synthase f0 subunit 6 |
| CUST_685_PI406199769 | combined_c345 | 10.13 | 5.43 | cytochrome c oxidase subunit i |
| CUST_1229_PI426302897 | Afun001229 | 9.54 | 7.41 | glucosyl glucuronosyl transferases |
| CUST_770_PI406199769 | combined_c387 | 9.48 | 6.43 | mitochondrial cytochrome c oxi. s. 5b isoform 1 |
