# Supplementary material for: Investigating molecular basis of lambda-cyhalothrin resistance in an Anopheles funestus population from Senegal
Source: Parasit Vectors. 2016 Aug 12;9:449. doi: 10.1186/s13071-016-1735-7 (PMC4983014; doi:10.1186/s13071-016-1735-7)
Supplement: Additional file 8: Table S8. — Top 50 the most detoxification genes under expressed in the R-S_L comparisons (FC ≥2, P ≤ 0.05). (DOCX 100 kb) [file 13071_2016_1735_MOESM8_ESM.docx]

**Table S8:** Top 50 the most detoxification genes under expressed in the **R-S_L** comparisons (FC ≥2, P≤ 0.05)

| **Probes Names** | **Transcripts** | **FC Abs R-S_L** | **Description** |
| --- | --- | --- | --- |
| CUST_13280_PI426302897 | Afun013280 | 29.52 | carboxypeptidase n subunit 2 |
| CUST_2375_PI406199772 | CD578215.1 | 24.47 | cuticle protein |
| CUST_5686_PI406199769 | combined_c2878 | 17.80 | nadh dehydrogenase |
| CUST_4339_PI406199772 | CD577197.1 | 17.72 | cytochrome c oxidase subunit iii |
| CUST_10482_PI426302897 | Afun010482 | 16.92 | cuticle protein |
| CUST_2748_PI406199772 | CD578027.1 | 16.29 | cytochrome c |
| CUST_2538_PI406199772 | CD578132.1 | 16.23 | pupal cuticle protein |
| CUST_4495_PI406199772 | CD577117.1 | 13.18 | atpase 6 |
| CUST_4508_PI406199772 | CD577111.1 | 13.02 | atp synthase f0 subunit 6 |
| CUST_7332_PI406199769 | combined_c3712 | 11.85 | stress-sensitive b |
| CUST_15633_PI406199769 | combined_c8299 | 11.43 | nadh dehydrogenase subunit 5 |
| CUST_5121_PI406199772 | BU038881 | 11.23 | h+ transporting atp synthase beta subunit |
| CUST_21_PI426302915 | CYP6S2.seq | 11.11 | cytochrome p450 |
| CUST_10684_PI426302897 | Afun010684 | 10.84 | juvenile hormone esterase |
| CUST_6003_PI406199769 | combined_c3036 | 10.49 | prophenoloxidase |
| CUST_3995_PI406199772 | CD577372.1 | 10.37 | cytochrome oxidase subunit i |
| CUST_16002_PI406199769 | combined_c8485 | 9.59 | cytochrome b |
| CUST_1229_PI426302897 | Afun001229 | 9.54 | glucosyl glucuronosyl transferases |
| CUST_770_PI406199769 | combined_c387 | 9.48 | mitochondrial cytochrome c oxidase subunit 5b isoform 1 |
| CUST_2690_PI406199772 | CD578056.1 | 9.11 | nadh dehydrogenase iron-sulfur protein mitochondrial |
| CUST_15359_PI426302897 | Afun015359 | 8.97 | para-nitrobenzyl esterase |
| CUST_13732_PI426302897 | Afun013732 | 8.48 | odorant binding protein (agap006080-pa) |
| CUST_9666_PI426302897 | Afun009666 | 8.29 | ankyrin repeat protein |
| CUST_12874_PI426302897 | Afun012874 | 7.97 | carboxylesterase 3 |
| CUST_14616_PI426302897 | Afun014616 | 7.54 | cytochrome p450 |
| CUST_14717_PI426302897 | Afun014717 | 7.34 | nadph oxidase |
| CUST_232_PI426302897 | Afun000232 | 6.82 | oxidase peroxidase |
| CUST_4173_PI406199772 | CD577280.1 | 6.78 | atp synthase lipid-binding mitochondrial precursor |
| CUST_15273_PI426302897 | Afun015273 | 6.78 | zinc carboxypeptidase |
| CUST_9117_PI426302897 | Afun009117 | 6.47 | cytochrome p450 |
| CUST_3687_PI406199772 | CD577539.1 | 6.37 | atp synthase subunit mitochondrial |
| CUST_2192_PI406199772 | CD578308.1 | 6.26 | nadh dehydrogenase flavoprotein mitochondrial |
| CUST_7604_PI406199798 | AGAP008955-RA | 6.14 | ubiquinol-cytochrome c reductase iron-sulfur subunit |
| CUST_4062_PI406199772 | CD577336.1 | 5.97 | cytochrome c oxidase subunit iv |
| CUST_3824_PI406199772 | CD577465.1 | 5.94 | mitochondrial cytochrome c oxidase subunit 7c |
| CUST_3024_PI406199772 | CD577888.1 | 5.93 | phosphoglyceromutase |
| CUST_15052_PI406199769 | combined_c8000 | 5.86 | cytochrome c oxidase subunit viia |
| CUST_1378_PI406199798 | AGAP001405-RA | 5.78 | short-chain dehydrogenase |
| CUST_354_PI406199788 | gb-CYP4G17 | 5.47 | cytochrome p450 |
| CUST_3804_PI406199772 | CD577475.1 | 5.30 | cytochrome c oxidase subunit va |
| CUST_10964_PI426302897 | Afun010964 | 5.29 | thioesterase superfamily member 2 |
| CUST_3620_PI406199772 | CD577573.1 | 5.28 | glutathione s-transferase |
| CUST_3468_PI406199772 | CD577653.1 | 5.11 | h+ transporting atp synthase subunit e |
| CUST_7911_PI426302897 | Afun007911 | 4.94 | glucosyl glucuronosyl transferase |
| CUST_9768_PI426302897 | Afun009768 | 4.86 | cuticular protein rr-1 family (agap009879-pa) |
| CUST_15227_PI426302897 | Afun015227 | 4.74 | cytochrome p450 |
| CUST_6_PI406199775 | CYP6AA4 | 4.63 | cytochrome p450 |
| CUST_14624_PI426302897 | Afun014624 | 4.29 | oxidoreductase |
| CUST_14927_PI426302897 | Afun014927 | 4.20 | esterase b1 |
| CUST_14618_PI426302897 | Afun014618 | 4.20 | odorant binding protein |
