# Supplementary material for: Investigating molecular basis of lambda-cyhalothrin resistance in an Anopheles funestus population from Senegal
Source: Parasit Vectors. 2016 Aug 12;9:449. doi: 10.1186/s13071-016-1735-7 (PMC4983014; doi:10.1186/s13071-016-1735-7)
Supplement: Additional file 9: Table S9. — Top 50 the most detoxification genes under expressed in the R-C_L comparisons (FC ≥1.5, P ≤ 0.05). (DOCX 95 kb) [file 13071_2016_1735_MOESM9_ESM.docx]

**Table S9:** Top 50 the most detoxification genes under expressed in the **R-C_L** comparisons (FC ≥1.5, P≤ 0.05)

| **Probes Names** | **Transcripts** | **FC Abs**  **R-C_L** | **Description** |
| --- | --- | --- | --- |
|  |  |  |  |
| CUST_1798_PI406199772 | EE589320.1 | 10.47 | mucin-like protein 60 |
| CUST_2644_PI406199772 | CD578079.1 | 9.08 | trypsin |
| CUST_11318_PI426302897 | Afun011318 | 7.99 | cuticular protein 144 (agap006369-pa) |
| CUST_10441_PI426302897 | Afun010441 | 7.68 | synaptic vesicle protein |
| CUST_8369_PI426302897 | Afun008369 | 7.67 | salivary protein |
| CUST_979_PI406199772 | EE589255.1 | 7.26 | sg3 protein |
| CUST_11294_PI426302897 | Afun011294 | 7.06 | peritrophic matrix protein 14 |
| CUST_7301_PI426302897 | Afun007301 | 7.02 | cytochrome p450 |
| CUST_714_PI406199772 | EE589506.1 | 6.06 | sg3 protein |
| CUST_700_PI406199772 | EE589520.1 | 5.94 | sg3 protein |
| CUST_14678_PI426302897 | Afun014678 | 5.32 | cd36 antigen |
| CUST_1267_PI406199772 | EE589890.1 | 5.10 | sg2 salivary protein |
| CUST_1057_PI406199772 | EE590117.1 | 4.76 | sg2 salivary protein |
| CUST_1619_PI406199772 | EE589514.1 | 4.28 | sg2 salivary protein |
| CUST_3482_PI406199769 | combined_c176 | 4.13 | Unknown |
| CUST_3483_PI406199769 | combined_c176 | 4.11 | Unknown |
| CUST_7630_PI426302897 | Afun007630 | 3.84 | conserved hypothetical protein [Cx quinquefasciatus] |
| CUST_2691_PI426302897 | Afun002691 | 3.81 | Unknown |
| CUST_121_PI426302897 | Afun000121 | 3.77 | Unknown |
| CUST_1849_PI406199772 | EE589262.1 | 3.69 | sg2 salivary protein |
| CUST_10005_PI426302897 | Afun010005 | 3.41 | alpha-amylase i |
| CUST_13707_PI426302897 | Afun013707 | 3.39 | cytochrome p450 |
| CUST_8603_PI426302897 | Afun008603 | 3.17 | cuticular protein rr-1 family (agap006009-pa) |
| CUST_11210_PI426302897 | Afun011210 | 3.09 | cationic amino acid transporter |
| CUST_8678_PI426302897 | Afun008678 | 2.90 | hairy |
| CUST_3551_PI426302897 | Afun003551 | 2.82 | fatty acid synthase |
| CUST_15374_PI406199769 | combined_c8168 | 2.77 | Unknown |
| CUST_15373_PI406199769 | combined_c8168 | 2.72 | Unknown |
| CUST_7418_PI426302897 | Afun007418 | 2.64 | sugar transporter |
| CUST_8876_PI406199769 | combined_c4496 | 2.52 | Unknown |
| CUST_9391_PI426302897 | Afun009391 | 2.51 | abc transporter |
| CUST_2586_PI426302897 | Afun002586 | 2.49 | pou domain drifter cf-1a |
| CUST_12935_PI426302897 | Afun012935 | 2.40 | pou domain drifter cf-1a |
| CUST_3372_PI406199769 | combined_c1704 | 2.34 | Unknown |
| CUST_7810_PI426302897 | Afun007810 | 2.30 | isoform a |
| CUST_9892_PI426302897 | Afun009892 | 2.17 | globin 1 |
| CUST_11340_PI426302897 | Afun011340 | 2.14 | atp-binding cassette transporter |
| CUST_8205_PI426302897 | Afun008205 | 2.14 | chymotrypsin 1 |
| CUST_9138_PI426302897 | Afun009138 | 2.13 | Unknown |
| CUST_7544_PI426302897 | Afun007544 | 2.12 | anopheles gambiae pest agap012703-pa |
| CUST_13601_PI426302897 | Afun013601 | 2.06 | odorant-binding protein |
| CUST_9547_PI426302897 | Afun009547 | 1.88 | niemann-pick type c- |
| CUST_7788_PI426302897 | Afun007788 | 1.79 | pantothenate kinase |
| CUST_14165_PI406199769 | combined_c7366 | 1.78 | Unknown |
| CUST_2560_PI406199769 | combined_c1292 | 1.76 | Unknown |
| CUST_7814_PI426302897 | Afun007814 | 1.70 | ribulose-phosphate 3-epimerase |
| CUST_10564_PI426302897 | Afun010564 | 1.68 | adenylate cyclase |
| CUST_3630_PI406199769 | combined_c1833 | 1.67 | Unknown |
| CUST_10664_PI426302897 | Afun010664 | 1.66 | malate dehydrogenase |
| CUST_3361_PI406199769 | combined_c1698 | 1.66 | tip41-like protein |
