# Supplementary figures and images for: Investigating molecular basis of lambda-cyhalothrin resistance in an Anopheles funestus population from Senegal
Source: Parasit Vectors. 2016 Aug 12;9:449. doi: 10.1186/s13071-016-1735-7 (PMC4983014; doi:10.1186/s13071-016-1735-7)

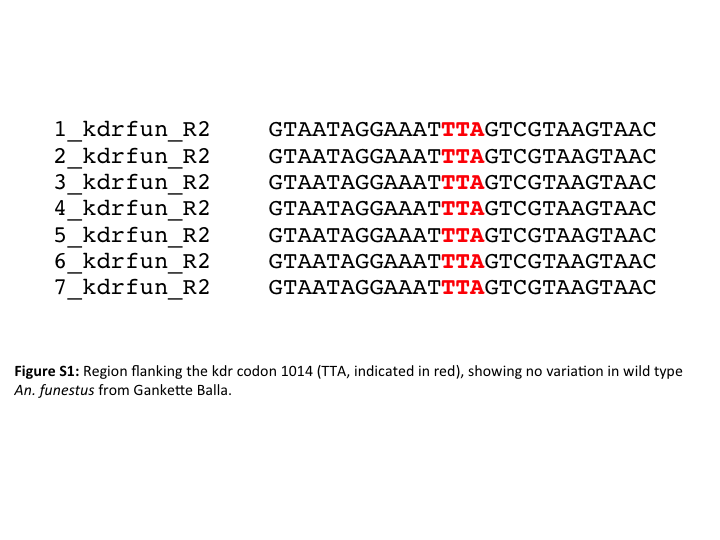

Supplement: Additional file 10: Figure S1. — Region flanking the kdr codon 1014 (TTA, indicated in red), showing no variation in wild type An. funestus from Gankette Balla. (TIF 1521 kb) [file 13071_2016_1735_MOESM10_ESM.tif]
